# Repurposing catheter ablation work-up to detect expiratory airflow limitation in patients with atrial fibrillation^☆^

**DOI:** 10.1016/j.ijcha.2023.101305

**Published:** 2023-11-17

**Authors:** Maartje J.M. Hereijgers, Rachel M.J. van der Velden, Nora el Moussaoui, Dominique V.M. Verhaert, Zarina Habibi, Justin Luermans, Dennis den Uijl, Sevasti-Maria Chaldoupi, Kevin Vernooy, Ulrich Schotten, Mathias Baumert, Hester A. Gietema, Casper Mihl, Lukasz Koltowski, Frits M.E. Franssen, Sami O. Simons, Dominik Linz

**Affiliations:** a Department of Cardiology, Maastricht University Medical Center, Maastricht, The Netherlands; b CARIM Cardiovascular Research Institute Maastricht, Maastricht University, Maastricht, The Netherlands; c Department of Cardiology, Radboud University Medical Center, Nijmegen, The Netherlands; d Department of Physiology, Cardiovascular Research Institute Maastricht, Maastricht University, Maastricht, The Netherlands; e Discipline of Biomedical Engineering, The University of Adelaide, Adelaide, Australia; f Department of Radiology and Nuclear Medicine, Maastricht University Medical Center, Maastricht, The Netherlands; g GROW School for Oncology and Developmental Biology, Maastricht University, Maastricht, The Netherlands; h First Department of Cardiology, Medical University of Warsaw, Warsaw, Poland; i Department of Research and Development, Ciro, Horn, The Netherlands; j Department of Respiratory Medicine, Maastricht University Medical Center, Maastricht, The Netherlands; k NUTRIM Research Institute of Nutrition and Translational Research in Metabolism, Maastricht University, Maastricht, The Netherlands; l Centre for Heart Rhythm Disorders, University of Adelaide and Royal Adelaide Hospital, Adelaide, Australia; m Department of Biomedical Sciences, Faculty of Health and Medical Sciences, University of Copenhagen, Copenhagen, Denmark

**Keywords:** Atrial fibrillation, Expiratory airflow limitation, Repurposing pre-ablation work-up, Cardiac computed tomographic angiography

## Abstract

**Background:**

In atrial fibrillation (AF) patients, presence of expiratory airflow limitation may negatively impact treatment outcomes. AF patients are not routinely screened for expiratory airflow limitation, but existing examinations can help identify at-risk individuals. We aimed to assess the diagnostic value of repurposing existing assessments from the pre-ablation work-up to identify and understand the characteristics of affected patients.

**Methods:**

We screened 110 consecutive AF patients scheduled for catheter ablation with handheld spirometry. Routine pre-ablation work-up included cardiac computed tomographic angiography (CCTA), transthoracic echocardiography and polygraphy. CCTA was analyzed qualitatively for emphysema and airway abnormalities. Multivariate logistic regression analysis was performed to determine predictors of expiratory airflow limitation.

**Results:**

We found that 25 % of patients had expiratory airflow limitation, which was undiagnosed in 86 % of these patients. These patients were more likely to have pulmonary abnormalities on CCTA, including emphysema (odds ratio [OR] 4.2, 95 % confidence interval [CI] 1.12–15.1, p < 0.05) and bronchial wall thickening (OR 2.6, 95 % CI 1.0–6.5, p < 0.05). The absence of pulmonary abnormalities on CCTA accurately distinguished patients with normal lung function from those with airflow limitation (negative predictive value: 85 %). Echocardiography and polygraphy did not contribute significantly to identifying airflow limitation.

**Conclusions:**

In conclusion, routine pre-ablation CCTA can detect pulmonary abnormalities in AF patients with airflow limitation, guiding further pulmonary assessment. Future studies should investigate its impact on ablation procedure success.

## 1 Introduction

Catheter-based pulmonary vein isolation (PVI) represents the cornerstone of modern rhythm control strategy in patients with symptomatic atrial fibrillation (AF) [1]. In addition to other established comorbidities such as sleep apnea and heart failure, the coexistence of expiratory airflow limitation not only amplifies morbidity and mortalitiy, but also hampers the effectiveness of rhythm control strategies. The most common causes of expiratory airflow limitation are chronic obstructive pulmonary disease (COPD) and bronchial asthma, and both are associated with an increased risk for AF [2], [3]. COPD is prevalent in up to 13 % and is accompanied by a two-fold higher risk of death and major bleeding [4], [5], [6]. Furthermore, COPD predicted higher risk of AF recurrence after ablation procedures [7].

The integrated Atrial Fibrillation Better Care (ABC) holistic pathway, as proposed in the guidelines of European Society of Cardiology (ESC), recommends the management of risk factors around PVI [1]. Recognizing expiratory airflow limitation as a frequent comorbidity and treatable risk factor may be important in this context. Nevertheless, no standardized recommendations on screening for expiratory airflow limitation are provided [8]. Spirometry is widely considered the primary diagnostic method for identifying expiratory airflow limitation [9]. However, conducting routine screenings for all AF patients may be time-consuming and expensive, while high-risk individuals may be identified from examinations that are part of work-up of AF patients. By utilizing existing resources, patients can undergo pulmonary screening without added burden on patients or the healthcare system [10], [11].

The objective of our study was to investigate whether certain characteristics obtained from standard pre-procedural cardiac imaging modalities, such as cardiac computed tomographic angiography (CCTA) and echocardiography, as well as oxygenation parameters derived from sleep apnea testing, can be used to identify AF patients with co-occuring expiratory airflow limitation.

## 2 Methods

### 2.1 Study design and study population

In the Maastricht University Medical Center (MUMC+), Maastricht, The Netherlands, consecutive symptomatic AF patients referred for catheter ablation undergo systematic screening for common comorbidities and triggers for AF, e.g. by handheld spirometry, sleep apnea testing, cardiac echocardiography and cardiac computed tomographic angiography (CCTA). In this context, all consecutive patients with symptomatic paroxysmal or persistent AF that completed the full pre-ablation work-up between August 2021 and March 2022 were prospectively enrolled in this study. AF was confirmed by at least one 12-lead electrocardiography (ECG) documentation in accordance with the ESC guidelines [1]. This study was part of the prospective ISOLATION cohort study (ClinicalTrials.gov identifier: NCT04342312), which is described in detail elsewhere [11]. The study was approved by the ethical review board MUMC+/Maastricht University [UM, NL number: 70787.068.19/METC number: 19-052] and complied with the Declaration of Helsinki. All participants gave written informed consent for use of their data.

### 2.2 Patient characteristics

Demographic and anthropometric data were collected at baseline during the first outpatient clinic visit. Medical history and associated treatment were retrieved from medical records. AF symptom burden was assessed with the European Heart Rhythm Association (EHRA) score, the Toronto AF severity scale questionnaire (AFSS) and the AF quality of life survey (AFEQT) [12], [13]. All patients were requested to fill out the STOP-Bang questionnaire to evaluate obstructive sleep apnea related symptoms at baseline [14]. Smoking status and presence of respiratory symptoms were retrieved from medical history, and in a subset AF quality of life survey (CAT) and modified medical research council dyspnea scale (mMRC) were taken^15^.

### 2.3 Handheld spirometry

In this study, two handheld spirometry devices were used to evaluate pulmonary function, either the Vitalograph COPD-6 (Vitalograph, Ireland) or AioCare® (HealthUp, Poland). In accordance with the European Respiratory Society guidelines, we defined expiratory airflow limitation as a pathological reduction in airflow from the lungs that leads to a reduced FEV1/FVC ratio [15], [16]. The spirometry equivalent of expiratory airflow limitation depended on the type of device used [17]. For Vitalograph COPD-6, expiratory airflow limitation was defined as forced expiratory volume in six seconds (FEV_6_) to forced vital capacity (FVC) ratio of 0.73 or less [18]. For AioCare, this was defined as forced expiratory volume in one second (FEV_1_) to FVC ratio of 0.70 or lower [19]. The severity of the expiratory airflow limitation was defined as the percentage predicted based on reference values and divided into mild (>80 %), moderate (50–80 %), severe (30–50 %) and very severe (<30 %) expiratory airflow limitation. Post-bronchodilator lung function testing, typically used for clinical characterization of conditions like asthma, COPD, or bronchiectasis, was not performed, as only prebronchodilator lung function testing was conducted.

### 2.4 Cardiac computed tomographic angiography

CCTAs were performed using a third-generation dual-source CT scanner (Somatom Definition Force, Siemens Healthineers, Forchheim Germany). Initially, a non-contrast enhanced scan was performed to evaluate the degree of coronary artery calcification (Agatston score [20] using semi-automatic post-processing software (syngo.via, Siemens Healthineers). This assessment was accomplished with reference milliampere-seconds (ref mAs) set at 80 and tube voltage at 120 kV (kV). This was followed by a CT angiography (Flash Care kV, rotation time 0.25 s, pitch 3.2, ref mAs 350, Kernel BV 36 image reconstruction). Patients received lopromide 300, an ionated contrast agent, tailored to their body weight. Prospective ECG triggering was used to synchronize the scan with the patient's heart's electrical activity, ensuring high-quality images with minimal motion artifacts. Image reconstruction was done with a 0.6 mm (mm) slice thickness with an increment of 0.4 mm. The field of view extends from the supra-aortic region to the heart base, encompassing parts of the thorax and upper abdomen. To evaluate the pulmonary field of view of a CCTA, two trained researchers (MH and NM) conducted a qualitative analysis for emphysema and airway abnormalities, supervised by an experienced radiologist using the COPD gene screening instrument [21]. Emphysema was visually scored as follows [22]: no emphysema, 1–5 % (trivial), 6–25 % (mild), 26–50 % (moderate), 51–75 % (severe) and > 75 % (very severe). If present, localization (i.e. upper, middle or lower lobe) and type of emphysema (i.e. centrilobular, paraseptal or panlobular) were evaluated. Airway abnormalities were visually scored based on bronchial wall thickening (i.e. absent, mild, severe), bronchial dilation and bronchiectasis (i.e. absent, present, equivocal, focal). The scores obtained from these various assessments were combined to create a comprehensive characterization of COPD based on radiologic features, including bronchial airway dominant, small airway dominant, emphysema dominant, mixed, and normal phenotypes. This classification covers various COPD phenotypes, including individuals without significant abnormalities.

### 2.5 Polygraphy

Screening for sleep apnea was performed using a home respiratory polygraphy device [23]. Patients with smartphones received the disposable WatchPAT-ONE, whereas patients without smartphones used the non-disposable WatchPAT 300 (Itamar Medical, Caesarea, Israel). Patients used the device for one night with a minimum valid recording time of 4 h. WatchPAT data was analyzed by a validated algorithm and reviewed by a certified sleep physician according to methods described in the American Academy of Sleep Medicine manual for scoring sleep and associated events [24]. Apnea-hypopnea index (AHI) was calculated as the total number of apneas plus hypopneas divided by the total sleep time in hours. The apnea severity was determined according to the following AHI categories: AHI 5–15, mild sleep apnea; AHI 15–30, moderate sleep apnea AHI ≥ 30, severe sleep apnea. The prevalence of clinically relevant sleep apnea was based on an AHI ≥ 15, aligning with treatment reimbursement criteria in patients with AF. The presence of symptoms were not included in this definition, as they have been shown to be unreliable in patients with atrial fibrillation [14]. To assess the value of home respiratory polygraphy for detecting patients with expiratory airflow limitation, the oximetry signals were further processed by a fully automated MATLAB-based computer algorithm, described in detail elsewhere [25]. This algorithm evaluated nocturnal hypoxemia burden defined as total sleep time spent at oxygen saturation levels below 90 % (T90). The oxygen desaturation index (ODI) was calculated as the number of desaturations of at least 4 % per hour of sleep. Furthermore, the duration of desaturation, nadirs, integral and amplitude of desaturation was calculated.

### 2.6 Echocardiography

Transthoracic echocardiography was performed according to a local protocol, which follows ESC practices [26], [27]. Left ventricular ejection fraction (LVEF) was preferably determined according to the 3D/Biplane method. If missing, LVEF was determined visually or via the Teichholz method. LVEF categories were set in line with the current consensus, i.e. ≤ 40 % equals reduced ejection fraction, 41–49 % equals mildly reduced ejection fraction, and ≥ 50 % equals preserved ejection fraction. Right ventricular systolic pressure (RVSP) was estimated based on peak tricuspid regurgitation velocity measured by continuous wave Doppler and right atrial pressure from inferior vena cava collapsibility. Left atrial (LA) and right atrial (RA) volumes were estimated from two-dimensional biplane quantitation.

### 2.7 Statistical analyses

All continuous variables were tested for normality with the Kolmogorov-Smirnov test and visual interpretation. Variables with normal distribution are expressed as mean ± standard deviation (SD) and non-parametric variables are expressed as median and interquartile range (IQR). Categorical data are presented as counts (n) with percentages (%). Fisher’s exact test (two groups) or χ2 test (three or more group comparisons) were used to compare categorical variables. Differences in continuous parameters were compared with unpaired *t*-test (two-group comparison) or ANOVA (three groups comparison) in the case of parametric variables and Mann–Whitney *U* test (two-group comparison) or Kruskal–Wallis test (three groups comparison) in the case of non-parametric variables.

To test the diagnostic value of routinely available studies from cardiac analysis, a multivariate logistic regression analysis with backward stepwise elimination was performed for all variables that reached significance in univariate analysis. Accuracy was expressed as sensitivity, specificity, and positive and negative predictive values of each diagnostic study (CCTA, echocardiography, polygraphy). The area under the curve (AUC) of receiver operating characteristic curves was assessed for each variable. A two-sided P value of 0.05 was considered statistically significant. Castor EDC (Electronic Data Capture, Amsterdam, The Netherlands) and IBM SPSS Version 28 (IBM Corporation, Somers, New York, USA) were used for database management and statistical analysis.

## 3 Results

### 3.1 Study population

A total of 110 consecutive patients scheduled for AF ablation were included in this study (70 % males; mean age 63 ± 10 years). The baseline characteristics of the study population are presented in Table 1.

**Table 1:** Baseline characteristics of the study population.

**Variables**	**Normal** (n = 82)	**Expiratory airflow** **limitation** (n = 28)	**p-value**
Demographics			
Age	64 (57–70)	69 (59–69)	0.12
Males	54 (66 %)	23 (82 %)	0.15
BMI (kg/m^2^)	28 ± 4.0	28 ± 3.8	1.00
Atrial fibrillation			
Paroxysmal	61 (74 %)	16 (57 %)	0.20
Persistent	21 (26 %)	12 (43 %)	
EHRA I – no symptoms	2 (2 %)	1 (4 %)	1.00
EHRA II – mild symptoms	63 (77 %)	21 (75 %)	
EHRA III – severe symptoms	17 (21 %)	6 (21 %)	
CHA_2_DS_2_-VASc score	1.5 (2.0)	2.0 (2.0)	0.67
CHA_2_DS_2_-VASc score ≥ 3 (female), ≥ 2 (male)	31 (38 %)	16 (57 %)	0.08
Medical history			
LVEF	10 (12 %)	7 (25 %)	0.13
≤40 %	3 (4 %)	0	
41–49 %	7 (9 %)	7 (25 %)	0.08
≥50 %	72 (88 %)	21 (75 %)	
Hypertension	31 (38 %)	17 (61 %)	**0.05**
Diabetes	7 (9 %)	0	0.19
Thromboembolic events	9 (13 % %)	1 (4 %)	0.28
Vascular disease	6 (9 %)	5 (18 %)	0.16
Hypercholesterolemia	18 (22 %)	8 (29 %)	0.61
Cardiovascular drugs			
0–1	5 (6 %)	3 (11 %)	0.11
2–3	53 (65 %)	12 (43 %)	
≥4	24 (29 %)	13 (46 %)	
Beta-blockers	43 (52 %)	11 (39 %)	0.28
Digitalis	3 (4 %)	3 (11 %)	0.17
Antiarrhythmic drugs	43 (52 %)	18 (64 %)	0.38
RAS inhibitors	30 (37 %)	16 (57 %)	0.12
Diuretics	9 (11 %)	3 (11 %)	1.00
CCB	4 (5 %)	3 (11 %)	0.37
Inhalation medication			
Single therapy (SABA)	2 (2 %)	0	
Dual therapy (LABA/ICS)	0	1 (1 %)	0.55^b^
Triple therapy (LABA/LAMA/ICS)	0	1 (1 %)	
Lifestyle factors			
Smoking			
Never	51 (62 %)	17 (61 %)	0.71
Current	15 (18 %)	5 (18 %)	
Former	12 (15 %)	6 (21 %)	
Alcohol			
None	17 (21 %)	7 (25 %)	0.93
<5 units/week	39 (48 %)	15 (54 %)	
5–15 units/week	14 (17 %)	1 (4 %)	
>15 units/week	5 (6 %)	1 (4 %)	
Symptom burden questionnaires			
AFSS	17 (12–24), *n = 73*	18 (11–24), *n = 23*	0.96
AFEQT	60 (50–76)	61 (48–77)	0.94
mMRC ≥ 2	7 (18 %), n = 39	1 (8 %), n = 12	0.66
CAT ≥ 10	8 (67 %), n = 12	9 (64 %), n = 12	1.00
Stop-BANG questionnaire	3 (2–4), n = 73	4 (3–5), n = 22	0.33
Spirometry characteristics			
FEV_1_/FEV_6_	0.84 (0.81–0.90), n = 50	0.58 (0.49–0.72), n = 14	<0.01
FEV_1_/FEV_6_ (%)	111 (105–117), n = 50	78 (58–95), n = 14	<0.01
FEV_1_/FVC	0.79 (0.76–0.82), n = 32	0.66 (0.63–0.70), n = 14	0.01
FEV_1_/FVC%	101 (97–106), n = 32	84 (82–90), n = 14	0.01
FVC (L)	4.4 (3.7–5.0), n = 32	4.1 (2.4–4.8), n = 14	0.22
FVC (%)	101 (87–114), n = 32	95 (85–104), n = 14	0.17
FEV_1_ (L)	3.0 ± 0.8, n = 82	2.1 ± 0.9, n = 28	<0.01
FEV_1_ (%)	96 (84–107), n = 82	66 (50–85), n = 28	<0.01
CCTA			
Emphysema			
No	30 (37 %)	14 (50 %)	**0.03^a^**
Trivial	25 (31 %)	8 (29 %)	
Mild	4 (5 %)	2 (7 %)	
Moderate	0	1 (4 %)	
Severe	1 (1 %)	3 (11 %)	
Bronchial wall thickening	39 (48 %)	18 (64 %)	**0.01**
Agatston calcium score	29 (0–231), *n = 72*	225 (3–389), *n = 26*	**0.05**
Polygraphy			
None-to-mild OSA (pAHI < 15)	38 (52 %), n = 73	15 (68 %), n = 22	0.23
Moderate-to-severe OSA (AHI ≥ 15)	35 (48 %), n = 73	7 (32 %), n = 22	

Abbreviations: BMI, body mass index; EHRA, European heart rhythm association score of atrial fibrillation; LVEF, left ventricular ejection fraction; RAS, renin–angiotensin system; CCB, calcium channel blocker; SABA, short-acting beta-agonist; LABA, long-acting beta-agonist; LAMA, long-acting muscarinic antagonist; ICS, inhalated corticosteroids; AFSS, atrial fibrillation symptom severity; AFEQT, atrial fibrillation quality of life survey; mMRC, modified medical research council; CAT, COPD assessment test; FEV1, forced expiratory volume in one second; FEV6, forced expiratory volume in six seconds; FVC, forced vital capacity; L, liter; CCTA, cardiac computed tomographic angiography; OSA, obstructive sleep apnea; AHI, apnea-hypopnea index.

Data are expressed as the median (interquartile range), mean ± standard deviation or number (percentage). Group differences were tested with the Mann-Whitney U and χ2 test.

^a^p-value corresponds with the statistical comparison of patients with none-to-trivial emphysema versus patients with mild-to-severe emphysema.

^b^p-value represents the statistical comparison between patients with inhalation medication and the patients without inhalation medication.

### 3.2 Spirometry

**Fig. 1:**
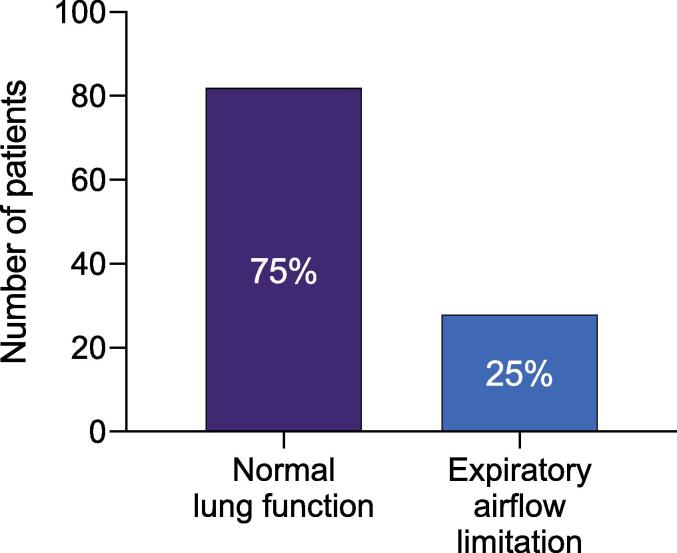
Prevalence of expiratory airflow limitation and normal lung function, *The y-axis represents the number of patients and the column displays the data as percentages.*

We found 24 patients with newly diagnosed expiratory airflow limitation and for an additional 4 patients a previous diagnosis was confirmed (in total 25 % of the study population; Fig. 1). Mild, moderate, severe and very severe expiratory airflow limitation were diagnosed in 10/110 (9 %), 11 (10 %), 5 (5 %) and 2 (2 %) patients, respectively. Spirometry characteristics of the study population are presented in Table 1. Out of the four patients known to have COPD, two patients used single therapy with exclusively short-acting beta-agonist medication, one patient used dual therapy of long-acting beta-agnostist (LABA) and inhaled corticosteroids (ICS), and one patient used triple therapy of long-acting muscarinic antagonist, LABA and ICS. Two self-reported COPD patients were categorized as having normal airflow; their mild airflow limitation and one patient's SABA use may have played a role in this classification. There was no correlation observed between expiratory airflow limitation and disease-specific symptom-severity questionnaires for AF, COPD, or sleep apnea.

### 3.3 Cardiac computed tomographic angiography

**Fig. 2:**
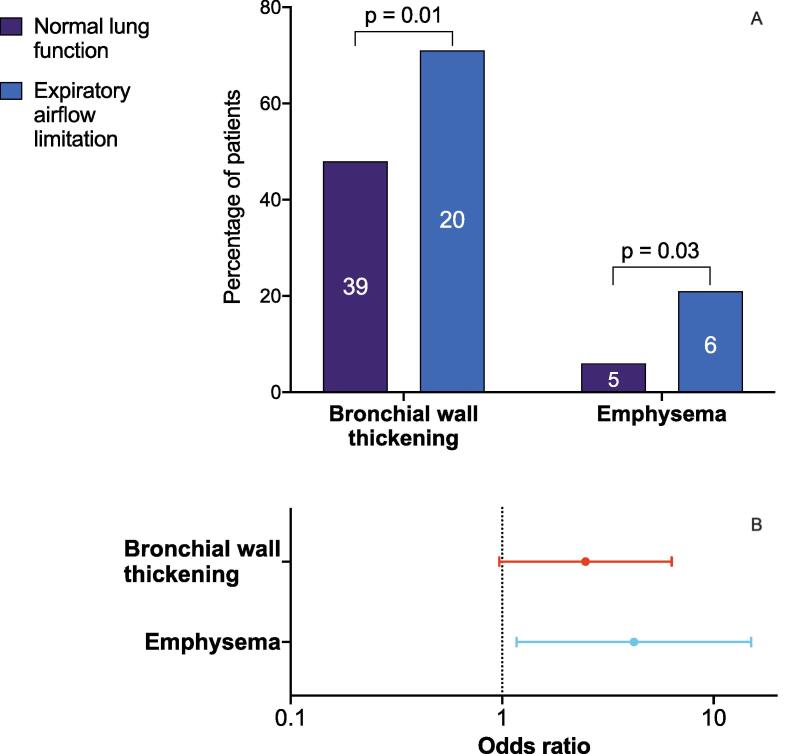
Pulmonary abnormalities from cardiac computed tomographic angiography and association with expiratory airflow limitation, A) Prevalence of pulmonary abnormalities from cardiac computed tomographic angiography in patients with and without expiratory airflow limitation. The y-axis represents the number of patients, while the column displays the data as percentages. B) Corresponding odds ratios and 95 % confidence intervals for the association with expiratory airflow limitation.

Emphysema was more prevalent in patients with expiratory airflow limitation (n = 6 [21 %]) than in patients with normal lung function (n = 5 [6 %]; p = 0.03; Fig. 2A). The extent of emphysema ranged from mild in 10 % to moderate in 7 % and severe in 4 % of all patients with expiratory airflow limitation. The prevalence of bronchial wall thickening (64 % vs. 48 %, p = 0.01) was significantly higher in patients with expiratory airflow limitation than in those with normal lung function. Agatston score of the coronary arteries was higher in patients with expiratory airflow limitation compared to those with normal lung function (225 [3–389] vs. 29 [0–231], p = 0.05). Expiratory airflow limitation was associated with mild-to-moderate emphysema (odds ratio [OR] 4.2, 95 % confidence interval [CI] 1.12–15.1) and bronchial wall thickening (OR 2.6, 95 % CI 1.0–6.5; Fig. 2B). Multivariate analyses demonstrated that these ORs remained largely independently associated with expiratory airflow limitation. Emphysema demonstrated a high specificity (i.e. 94 % and 91 %, respectively) with a negative predictive value of 78 %. For the phenotype score, the negative predictive value increased to 85 %. The AUCs to detect expiratory airflow limitation were 0.58 (95 % CI 0.45–0.71) for emphysema and 0.64 (95 % CI 0.52–0.76) for bronchial wall thickening, and 0.63 (95 % CI 0.51–0.74) for the phenotype score.

### 3.4 Polygraphy

**Fig. 3:**
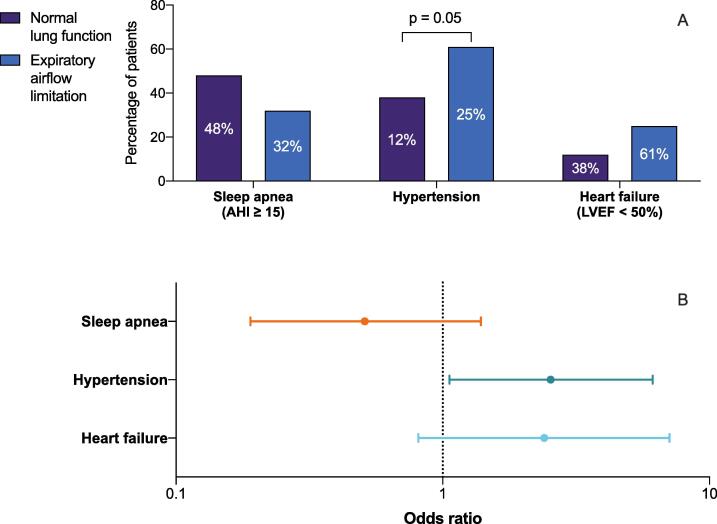
Prevalence of sleep apnea, heart failure, and hypertension in patients with expiratory airflow limitation, A) Prevalence of sleep apnea, heart failure, and hypertension in patients with and without airflow limitation. The y-axis represents the number of patients, while the column displays the data as percentages. B) Corresponding odds ratios and 95 % confidence interval for the association with expiratory airflow limitation.

The prevalence of moderate-to-severe sleep apnea was 32 % among individuals with expiratory airflow limitation and 48 % among individuals with normal lung function (p = 0.23; Fig. 3). Mild, moderate and severe sleep apnea were newly diagnosed in 11 (50 %), 4 (18 %) and 3 (14 %) patients with expiratory airflow limitation, respectively (Supplementary Table S1). For patients with normal lung function, this comprised 31 (43 %) patiens with mild, 26 (36 %) with moderate and 9 (11 %) with severe sleep apnea. Fourteen patients had no results available (5 had not returned the device yet, 4 refused screening, 2 had pending results, 2 for unknown reasons, 1 had unsuccessful measurements). T90, calculated by the additional nocturnal hypoxemia algorithm, corresponded with 0.5 (0.1–2.5) minutes in patients with expiratory airflow limitation and 1.2 (0.2–2.5) minutes in patients with normal lung function (p = 0.46; Supplementary Table S2 and SupplementaryTable S3. Further characterization from the additional nocturnal hypoxemia algorithm showed a significantly lower ODI in patients with expiratory airflow limitation than in those with normal lung function (1.4 [0.8–2.1] vs. 3.4 [1.3–3.6], p = 0.01). Accordingly, the total number of nocturnal desaturations were significantly lower in patients with expiratory airflow limitation (10 [6], [7], [8], [9], [10], [11], [12], [13], [14], [15], [16], [17] vs. 26 [10–51], p = 0.01). No significant differences were found for duration, integral, amplitude or nadir of desaturation. The number of desaturations and ODI were associated with a lower risk of expiratory airflow limitation (OR 0.98 [0.95–1.00], p = 0.052; OR 0.86 [0.73–1.02], p = 0.078). AUCs to detect expiratory airflow limitation were 0.69 (95 % CI 0.56–0.82) for ODI and 0.69 (95 % CI 0.56–0.82) for the number of desaturations.

### 3.5 Echocardiography

Reduced LVEF was prevalent in patients with and without expiratory airflow limitation (25 % vs 12 %, p = 0.08). No difference in RVSP was found between patients with expiratory airflow limitation versus patients with normal lung function (25 [11], [12], [13], [14], [15], [16], [17], [18], [19], [20], [21], [22], [23], [24], [25], [26], [27], [28], [29], [30] and 25 [20], [21], [22], [23], [24], [25], [26], [27], [28], [29], [30] mmHg, respectively). Elevated RVSP (i.e. > 35 mmHg) was present in only three patients of the total study population (i.e. two patients with normal lung function and one patient with expiratory airflow limitation). Therefore, no further analyses were conducted. LA and RA volumes were also equally distributed for patients with expiratory airflow limitation and normal lung function (LA: 77 [52–94] mL and 76 [59–92] mL, p = 0.95; RA: 54 [40–70] mL and 53 (37–71] mL, p = 0.57). The results obtained from echocardiography are presented in Supplementary Table S4.

### 3.6 Comorbidities

Hypertension was more often prevalent in patients with expiratory airflow limitation compared to patients with normal lung function (61 % vs. 38 %, p = 0.05). The likelihood of expiratory airflow limitation was higher in patients with hypertension (OR 2.5, [95 CI 1.1–6.1], p = 0.04) and in patients with higher thromboembolic risk (CHA_2_DS_2_-VASc score ≥ 3 female or ≥ 2 male 57 % vs. 38 %, p = 0.08) compared to patients with a normal lung function.

## 4 Discussion

This study confirms that expiratory airflow limitation is common and frequently overlooked in AF patients scheduled for ablation. Signs of COPD present at CCTA can be used to identify patients with expiratory airflow limitation, while lack thereof is highly specific to exclude patients. This novel insight has the potential to utilize existing resources from the pre-ablation work-up to facilitate spirometry prioritization. The other available resources were not contributing to detect expiratory airflow limitation.

We tested the hypothesis that available pre-procedural CCTA can be utilized to identify and understand the characteristics of patients with expiratory airflow limitation. Our results showed that patients with emphysema and bronchial wall thickening had a 2.5–4.2-fold higher risk of expiratory airflow limitation. Emphysema was highly specific (95 % and 91 %, respectively) to differentiate patients with normal lung function from those with expiratory airflow limitation. However, the sensitivity of CCTA to detect expiratory airflow limitation was lower. This can be attributed to two factors, namely, the predilection site for centrilobular emphysema in the apical lung fields, which is not adequately captured in the limited pulmonary view provided by CCTA, and the limited involvement of lung parenchyma in patients with asthma [28]. Moreover, it is important to note that 60 % of our study population consists of individuals who have never smoked, which inherently reduces the initial likelihood of discovering emphysema or airway abnormalities. Furthermore, among patients scheduled for AF ablation procedures, it should be considered that the coexisting pulmonary disease may not be in an advanced stage, and early signs of COPD or asthma may not be detectable on CCTA at this point. Software-assisted evaluation has the potential to improve the detection and assessment of emphysema, enhancing the accuracy and efficiency of diagnostic processes in the future [22]. Although the evaluation of emphysema using software assistance was not feasible in this study due to the utilization of contrast-enhanced CT scans, it presents an intriguing possibility for future perspectives.

Regarding the other available resources of the pre-ablation work-up, echocardiography was insufficient to detect patients with expiratory airflow limitation. The RVSP, the echocardiographic estimation of pulmonary hypertension, may be elevated in patients with severe chronic lung disease due to hypoxic pulmonary vasoconstriction [29], [30]. The present study population predominantly presented with mild-to-moderate expiratory airflow limitation and emphysema. Therefore, the poor discriminating performance of echocardiography parameters might be explained by the relatively low prevalence of severe lung disease within this study population. In addition, previous studies also found a very low diagnostic accuracy of RVSP to detect pulmonary hypertension in patients with emphysema [31]. The implementation of more innovative technologies utilizing speckle tracking echocardiography to assess atrial function has the potential to bring about significant advancements [32].

One unanticipated finding of the present study was that the nocturnal hypoxemic burden was less severe in patients with expiratory airflow limitation. This rather paradoxical result may be because patients with expiratory airflow limitation, specifically COPD, have less collapsible airways due to air trapping. This has been previously described in COPD patients [33], [34], yet does not seem as apparent in asthma patients [35], [36]. Additionaly, the prevalence of sleep apnea was higher in patients with normal lung function, potentially resulting in increased nocturnal hypoxemic burden due to consecutive apnea-related desaturations.

In this study, systematic lung function testing revealed previously undiagnosed expiratory airflow limitation in a substantial proportion (22 %) of patients undergoing AF catheter ablation. The prevalence of expiratory airflow limitation is higher than previously reported numbers from pooled data [4], which may be attributable to the fact that the prevalence of asthma was not incorporated in this meta-analysis. Furthermore, we observed patients with expiratory airflow limitation tended to have a higher prevalence of hypertension and heart failure, yielding a heightened thromboembolic risk as determined by the CHA2DS2-VASc score. This aligns with the results of the previously mentioned meta-analysis in AF patients with concomitant expiratory airflow limitation due to COPD [4].

No correlation was found between expiratory airflow limitation and symptom severity questionnaires for AF, COPD or sleep apnea. These findings indicate that relying solely on disease-specific questionnaires to assess symptoms may not be enough to rule out the presence of co-existing expiratory airflow limitation in patients with AF. Similar limitations are observed when using these questionnaires for other pulmonary comorbidities in AF patients, such as sleep apnea [14], [37], [38]. This may be explained by the shared risk factors and overlapping symptoms between AF and pulmonary comorbidities [14]. Also in the general population, the effectiveness of symptom- and risk factor-based questionnaires in screening for COPD has been found to be insufficient, highlighting their limited utility [39], [40].

We acknowledge the variability in standard diagnostic practices, which may not be universal in all centers. However, our research underscores the potential of emerging techniques and the need for optimization. We aim to streamline pre-ablation work-ups based on available resources to enhance overall AF management. Our findings on a COPD screening and management pathway suggest that embedding such pathways, utilizing (micro)spirometry and remote result analysis, is feasible within an existing AF outpatient clinic infrastructure [41]. This, along with available resources, holds promise for enhancing spirometry prioritization and streamlining the diagnostic process for AF and comorbid conditions, ultimately contributing to more effective and efficient patient care. In this context, our study results emphasize the currently underutilized potential to detect expiratory airflow limitation using CCTA within the routine pre-procedural work-up in AF patients scheduled for catheter ablation. Although the present study did not directly examine the topic, it is important to consider the practical implications that arise from these findings. One such implication is the opportunity of close multidisciplinary collaboration among cardiologists, pulmonologists, and radiologists in the management of AF. To effectively facilitate this collaboration, an integrated care approach with regular multidisciplinary meetings may prove to be the most beneficial. By embracing such an integrated approach, healthcare professionals can enhance patient care and improve outcomes through effective teamwork and the utilization of diverse perspectives.The need and support for an integrated multidisciplinary approach to AF care, including pulmonary diseases, was also highlighted through a recent pan-European EHRA-PATHS member survey [42].

### 4.1 Study limitations

While the study is limited by a small sample size and the absence of a control group, the external validity of the study is considered satisfactory because the overall cohort exhibits representative baseline characteristics. However, the absence of a control group leaves the potential benefits of pulmonary screening on the success of the ablation procedure uncertain. Secondly, our study involved the use of two different spirometry devices. Although this choice is unlikely to have significantly influenced the core evaluation of expiratory airflow limitation, it may have had an impact on the comparability of severity levels between these devices. Additionaly, we only performed pre-bronchodilator measurements, which may not be sufficient for confirming COPD or asthma diagnoses, as post-bronchodilator measurements are required. Moreover, unpublished observations highlighted differences in accuracy between the devices, specifically noting the low sensitivity of the COPD-6 device [43]. Consequently, this discrepancy could result in elevated rates of false negatives, leading to an underestimation of expiratory airflow limitation in the patients tested with the COPD-6 device.

### 4.2 Conclusions

In conclusion, patients with expiratory airflow limitation more often present with CCTA-derived pulmonary abnormalities. This knowledge presents an opportunity to repurpose existing resources from the pre-ablation work-up, aiding in the pre-selection process of AF patients who need formal lung function screening. The clinical performance and the potential benefits on the success of the ablation procedure needs to be investigated in future studies.

## Declaration of competing interest

The authors declare the following financial interests/personal relationships which may be considered as potential competing interests: [US received grants, consultancy fees or honoraria from Università della Svizzera Italiana (USI, Switzerland), EP Solutions Inc. (Switzerland), Johnson & Johnson Medical Limited, (United Kingdom), Bayer Healthcare (Germany). US is co-founder and shareholder of YourRhythmics BV, a spin-off company of the University Maastricht.].

## References

[1] Hindricks G., Potpara T., Dagres N., Arbelo E., Bax J.J., Blomstrom-Lundqvist C. (2021). 2020 ESC Guidelines for the diagnosis and management of atrial fibrillation developed in collaboration with the European Association for Cardio-Thoracic Surgery (EACTS): The Task Force for the diagnosis and management of atrial fibrillation of the European Society of Cardiology (ESC) Developed with the special contribution of the European Heart Rhythm Association (EHRA) of the ESC. Eur. Heart J..

[2] Grymonprez M., Vakaet V., Kavousi M., Stricker B.H., Ikram M.A., Heeringa J. (2019). Chronic obstructive pulmonary disease and the development of atrial fibrillation. Int. J. Cardiol..

[3] Tattersall M.C., Dasiewicz A.S., McClelland R.L., Gepner A.D., Kalscheur M.M., Field M.E. (2020). Persistent Asthma Is Associated With Increased Risk for Incident Atrial Fibrillation in the MESA. Circ. Arrhythm Electrophysiol..

[4] Romiti G.F., Corica B., Pipitone E., Vitolo M., Raparelli V., Basili S. (2021). Prevalence, management and impact of chronic obstructive pulmonary disease in atrial fibrillation: a systematic review and meta-analysis of 4,200,000 patients. Eur. Heart J..

[5] Ye J., Yao P., Shi X., Yu X. (2022). A systematic literature review and meta-analysis on the impact of COPD on atrial fibrillation patient outcome. Heart Lung.

[6] Pisters R., Nieuwlaat R., Prins M.H., Le Heuzey J.Y., Maggioni A.P., Camm A.J. (2012). Clinical correlates of immediate success and outcome at 1-year follow-up of real-world cardioversion of atrial fibrillation: the Euro Heart Survey. Europace..

[7] Gu J., Liu X., Tan H., Zhou L., Jiang W., Wang Y. (2013). Impact of chronic obstructive pulmonary disease on procedural outcomes and quality of life in patients with atrial fibrillation undergoing catheter ablation. J. Cardiovasc. Electrophysiol..

[8] Simons S.O., Elliott A., Sastry M., Hendriks J.M., Arzt M., Rienstra M. (2021). Chronic obstructive pulmonary disease and atrial fibrillation: an interdisciplinary perspective. Eur. Heart J..

[9] GLOBAL INITIATIVE FOR CHRONIC OBSTRUCTIVE LUNG DISEASE. Global strategy for the diagnosis, management, and prevention of chronic obstructive pulmonary disease (2023 report). 2023.

[10] Verhaert D.V.M., Linz D., Wassink G.F., Weijs B., Philippens S., Luermans J. (2022). A new efficient and integrated pathway for patient evaluation prior to atrial fibrillation ablation. Eur. J. Cardiovasc. Nurs..

[11] Verhaert D.V.M., Linz D., Chaldoupi S.M., Westra S.W., den Uijl D.W., Philippens S. (2022). Rationale and Design of the ISOLATION Study: A Multicenter Prospective Cohort Study Identifying Predictors for Successful Atrial Fibrillation Ablation in an Integrated Clinical Care and Research Pathway. Front. Cardiovasc. Med..

[12] Dorian P., Mangat I. (2003). Quality of life variables in the selection of rate versus rhythm control in patients with atrial fibrillation: observations from the Canadian Trial of Atrial Fibrillation. Card Electrophysiol. Rev..

[13] Spertus J., Dorian P., Bubien R., Lewis S., Godejohn D., Reynolds M.R. (2011). Development and validation of the Atrial Fibrillation Effect on QualiTy-of-Life (AFEQT) Questionnaire in patients with atrial fibrillation. Circ. Arrhythm. Electrophysiol..

[14] Delesie M., Knaepen L., Hendrickx B., Huygen L., Verbraecken J., Weytjens K. (2021). The value of screening questionnaires/scoring scales for obstructive sleep apnoea in patients with atrial fibrillation. Arch. Cardiovasc. Dis..

[15] Singh D., Agusti A., Anzueto A., Barnes P.J., Bourbeau J., Celli B.R. (2019). Global Strategy for the Diagnosis, Management, and Prevention of Chronic Obstructive Lung Disease: the GOLD science committee report 2019. Eur. Respir. J..

[16] Graham B.L., Steenbruggen I., Miller M.R., Barjaktarevic I.Z., Cooper B.G., Hall G.L. (2019). Standardization of Spirometry 2019 Update. An Official American Thoracic Society and European Respiratory Society Technical Statement. Am. J. Respir. Crit. Care Med..

[17] Stanojevic S., Kaminsky D.A., Miller M.R., Thompson B., Aliverti A., Barjaktarevic I. (2022). ERS/ATS technical standard on interpretive strategies for routine lung function tests. Eur. Respir. J..

[18] Dickens A.P., Fitzmaurice D.A., Adab P., Sitch A., Riley R.D., Enocson A. (2020). Accuracy of Vitalograph lung monitor as a screening test for COPD in primary care. NPJ Prim Care Respir. Med..

[19] Boros P.W., Maciejewski A., Nowicki M.M., Wesolowski S. (2022). Comparability of portable and desktop spirometry: a randomized, parallel assignment, open-label clinical trial. Adv. Respir. Med..

[20] Agatston A.S., Janowitz W.R., Hildner F.J., Zusmer N.R., Viamonte M., Detrano R. (1990). Quantification of coronary artery calcium using ultrafast computed tomography. J. Am. Coll Cardiol..

[21] Lowe K.E., Regan E.A., Anzueto A., Austin E., Austin J.H.M., Beaty T.H. (2019). COPDGene((R)) 2019: Redefining the Diagnosis of Chronic Obstructive Pulmonary Disease. Chronic Obstr. Pulm. Dis..

[22] Gietema H.A., Muller N.L., Fauerbach P.V., Sharma S., Edwards L.D., Camp P.G. (2011). Quantifying the extent of emphysema: factors associated with radiologists' estimations and quantitative indices of emphysema severity using the ECLIPSE cohort. Acad. Radiol..

[23] Verhaert D.V.M., Betz K., Gawalko M., Hermans A.N.L., Pluymaekers N., van der Velden R.M.J. (2022). A VIRTUAL Sleep Apnoea management pathway For the work-up of Atrial fibrillation patients in a digital Remote Infrastructure: VIRTUAL-SAFARI. Europace..

[24] Berry R.B., Brooks R., Gamaldo C., Harding S.M., Lloyd R.M., Quan S.F. (2017). AASM Scoring Manual Updates for 2017 (Version 2.4). J. Clin. Sleep Med..

[25] Linz D., Kadhim K., Brooks A.G., Elliott A.D., Hendriks J.M.L., Lau D.H. (2018). Diagnostic accuracy of overnight oximetry for the diagnosis of sleep-disordered breathing in atrial fibrillation patients. Int. J. Cardiol..

[26] M. Authors/Task Force, T.A. McDonagh, M. Metra, M. Adamo, R.S. Gardner, A. Baumbach, et al. 2021 ESC Guidelines for the diagnosis and treatment of acute and chronic heart failure: Developed by the Task Force for the diagnosis and treatment of acute and chronic heart failure of the European Society of Cardiology (ESC). With the special contribution of the Heart Failure Association (HFA) of the ESC. Eur J Heart Fail. 2022;24(1):4-131. 10.1002/ejhf.2333 35083827

[27] L.G. Rudski, W.W. Lai, J. Afilalo, L. Hua, M.D. Handschumacher, K. Chandrasekaran, et al. Guidelines for the echocardiographic assessment of the right heart in adults: a report from the American Society of Echocardiography endorsed by the European Association of Echocardiography, a registered branch of the European Society of Cardiology, and the Canadian Society of Echocardiography. J Am Soc Echocardiogr. 2010;23(7):685-713; quiz 86-8. 10.1016/j.echo.2010.05.010 20620859

[28] Global Initiative for Asthma. Global Strategy for Asthma Management and Prevention. Available from: www.ginasthma.org; 2022.

[29] J.R. Klinger, Group III Pulmonary Hypertension: Pulmonary Hypertension Associated with Lung Disease: Epidemiology, Pathophysiology, and Treatments. Cardiol Clin. 2016;34(3):413-33. 10.1016/j.ccl.2016.04.003 27443138

[30] Humbert M., Kovacs G., Hoeper M.M., Badagliacca R., Berger R.M.F., Brida M. (2022). 2022 ESC/ERS Guidelines for the diagnosis and treatment of pulmonary hypertension. Eur. Respir. J..

[31] Minai O.A., Fessler H., Stoller J.K., Criner G.J., Scharf S.M., Meli Y. (2014). Clinical characteristics and prediction of pulmonary hypertension in severe emphysema. Respir Med..

[32] van Mourik M.J.W., Artola Arita V., Lyon A., Lumens J., De With R.R., van Melle J.P. (2022). Association between comorbidities and left and right atrial dysfunction in patients with paroxysmal atrial fibrillation: Analysis of AF-RISK. Int. J. Cardiol..

[33] P. Biselli, P.R. Grossman, J.P. Kirkness, S.P. Patil, P.L. Smith, A.R. Schwartz, et al. The effect of increased lung volume in chronic obstructive pulmonary disease on upper airway obstruction during sleep. J Appl Physiol (1985). 2015;119(3):266-71. 10.1152/japplphysiol.00455.2014 PMC4526705 26048975

[34] Heinzer R.C., Stanchina M.L., Malhotra A., Jordan A.S., Patel S.R., Lo Y.L. (2006). Effect of increased lung volume on sleep disordered breathing in patients with sleep apnoea. Thorax.

[35] Sundbom F., Janson C., Ljunggren M., Lindberg E. (2022). Asthma and asthma-related comorbidity: effects on nocturnal oxygen saturation. J. Clin. Sleep Med..

[36] Higham M.A., Dawson D., Joshi J., Nihoyannopoulos P., Morrell N.W. (2001). Utility of echocardiography in assessment of pulmonary hypertension secondary to COPD. Eur. Respir. J..

[37] Kadhim K., Middeldorp M.E., Elliott A.D., Jones D., Hendriks J.M.L., Gallagher C. (2019). Self-Reported Daytime Sleepiness and Sleep-Disordered Breathing in Patients With Atrial Fibrillation: SNOozE-AF. Can. J.Cardiol..

[38] Betz K., Verhaert D.V.M., Gawalko M., Hermans A.N.L., Habibi Z., Pluymaekers N. (2023). Atrial fibrillation-specific refinement of the STOP-Bang sleep apnoea screening questionnaire: insights from the Virtual-SAFARI study. Clin. Res. Cardiol..

[39] Force U.S.P.S.T., Mangione C.M., Barry M.J., Nicholson W.K., Cabana M., Caughey A.B. (2022). Screening for Chronic Obstructive Pulmonary Disease: US Preventive Services Task Force Reaffirmation Recommendation Statement. J. Am. Med. Assoc..

[40] Guirguis-Blake J.M., Senger C.A., Webber E.M., Mularski R.A., Whitlock E.P. (2016). Screening for Chronic Obstructive Pulmonary Disease: Evidence Report and Systematic Review for the US Preventive Services Task Force. J. Am. Med. Assoc..

[41] van der Velden R.M.J., Hereijgers M.J.M., Arman N., van Middendorp N., Franssen F.M.E., Gawalko M. (2023). Implementation of a screening and management pathway for chronic obstructive pulmonary disease in patients with atrial fibrillation. Europace..

[42] Lee G., Baker E., Desteghe L., Heidbuchel H., Collins R., Merino J.L. (2022). The challenge of managing multimorbid atrial fibrillation: a pan-European European Heart Rhythm Association (EHRA) member survey of current management practices and clinical priorities. Europace.

[43] R.M.J. Van der Velden, N. Arman, M.J.M. Hereijgers, M. Gawalko, D.V.M. Verhaert, Z. Habibi, et al. Accuracy of two handheld (micro)spirometers in assessing lung function in patients with atrial fibrillation and dyspnoea: a comparative study. [Manuscript submitted for publication]. 2023.

