# Supplementary material for: Repurposing catheter ablation work-up to detect expiratory airflow limitation in patients with atrial fibrillation
Source: Int J Cardiol Heart Vasc. 2023 Nov 17;49:101305. doi: 10.1016/j.ijcha.2023.101305 (PMC10694302; doi:10.1016/j.ijcha.2023.101305)
Supplement: Supplementary data 1 [file mmc1.docx]

**Supplementary Table S*1*.** Sleep characteristics from overnight oximetry in patients with normal lung function and expiratory airflow limitation

| **Sleep characteristics** | **Normal** (n = 73) | **Expiratory airflow limitation** (n = 22) | **p-value** |
| --- | --- | --- | --- |
|  |  |  |  |
| Sleep architecture |  |  |  |
| Recording time (min) | 477 ± 60 | 477 ± 47 | 0.99 |
| Sleep time (min) | 402 ± 61 | 414 ± 47 | 0.44 |
| Sleep latency (min) | 20 (10-26) | 20 (15-25) | 0.91 |
| Awakenings | 9 (7-13) | 9 (5-11) | 0.12 |
| Sleep study results |  |  |  |
| pRDI | 18 (11-26), n=72 | 13 (9-28) | 0.29 |
| pAHI | 15 (8-22) | 10 (8-15) | 0.12 |
| Mean SpO2 (%) | 94 (93-95), n=72 | 94 (92-95) | 0.33 |
| Minimal SpO2 (%) | 86 (84-88), n=72 | 87 (83-90) | 0.50 |
| Maximal SpO2 (%) | 98 (98-99), n = 71 | 98 (97-99) | 0.19 |
| Pulse rate statistics |  |  |  |
| Mean (b.p.m.) | 58 (52-65) | 64 (54-68) | 0.22 |
| Maximal (b.p.m.) | 90 (82-104) | 96 (83-111) | 0.42 |
| SDB categories |  |  |  |
| No (pAHI < 5) | 7 (10%) | 4 (18%) | 0.36 |
| Mild (pAHI 5 to <15) | 31 (43%) | 11 (50%) |  |
| Moderate (pAHI 15 to <30) | 26 (36%) | 4 (18%) |  |
| Severe (pAHI>30) | 9 (12%) | 3 (14%) |  |

Note: Data are expressed as the median (interquartile range), mean ± standard deviation or number (percentage). Group differences were tested with the Mann-Whitney U and χ2 test.

Abbreviations: pRDI, respiratory disturbance index; AHI, apnea-hypopnea index; b.p.m., beats per minute.

**Supplementary Table S*2*.** Desaturation variables from overnight polygraphy in patients with normal lung function and expiratory airflow limitation

| **Nocturnal hypoxemic burden** | **Normal** (n = 71) | **Expiratory airflow limitation** (n = 22) | **p-value** |
| --- | --- | --- | --- |
|  |  |  |  |
| **T90 statistics** |  |  |  |
| T90 total (min) | 1.2 (0.2-2.5) | 0.5 (0.1-2.5) | 0.46 |
| T90 total normalized (% of sleeping time) | 0.3 (0.0-0.6) | 0.1 (0.0-0.3) | 0.40 |
| **Desaturation characteristics** |  |  |  |
| Total number of desaturations | 26 (10-51) | 10 (6-17) | **0.01** |
| Oxygen desaturation index | 3.4 (1.3-6.3) | 1.4 (0.8-2.1) | **0.01** |
| Median duration | *37 (33-47), n=69* | *36 (32-61), n=21* | 0.67 |
| Median integral | *90 (74-115), n=69* | *78 (69-140), n=21* | 0.61 |
| Median amplitude | *2.4 (2.2-2.7), n=69* | *2.2 (2.1-2.5), n=21* | 0.15 |
| Medan nadir | *91 (90-92), n=69* | *91 (90-92), n=21* | 0.43 |

Note: Data are expressed as the median (interquartile range). Group differences were tested with the Mann-Whitney U test.

Abbreviations: T90, minutes spent in peripheral capillary oxygen saturation below 90%.

**Supplementary Table S3**. Odds ratios of univariate logistic regression analysis to detect expiratory airflow limitation

| **Nocturnal hypoxemic burden** | **Univariate analyses** | | | | |
| --- | --- | --- | --- | --- | --- |
|  | **OR** | **95% CI** | | **p-value** | **AUC** |
|  |  | Lower | Upper |  |  |
| Number of desaturations (per night) | 0.98 | 0.95 | 1.00 | 0.05 | 0.69 |
| Oxygen desaturation index | 0.86 | 0.73 | 1.02 | 0.08 | 0.69 |

Note: Data are expressed as odds ratios with accompanying 95% confidence intervals and p-value, and area under the curve of receiver operating characteristic curves.

Abbreviations: OR, Odds ratio; CI, confidence interval; AUC, area under the curve of receiver operating characteristic curve.

**Supplementary Table S*4***. Echocardiography characteristics in patients with normal lung function and expiratory airflow limitation

| **Characteristics** | **Normal**  (n = 82) | **Expiratory airflow limitation** (n = 28) | **p-value** |
| --- | --- | --- | --- |
| RVSP (mmHg) | 25 (11-30) | 25 (20-30) | 0.95 |
| RVSP>40mmHg | 2 (2%) | 1 (4%) | 1.00 |
| LVEF (%) | 57 (54-61) | 57 (50-60) | 0.51 |
| LVEF categories |  |  |  |
| ≤40% | 3 (4%) | 0 |  |
| 41-49% | 7 (9%) | 7 (25%) | 0.08 |
| ≥50% | 72 (88%) | 21 (75%) |  |
| LA-volume (mL) | 76 (59-92) | 77 (52-94) | 0.95 |
| RA-volume (mL) | 53 (37-71) | 54 (40-70) | 0.57 |

Note: Data are expressed as the median (interquartile range) or number (percentage). Group differences were tested with the Mann-Whitney U and χ2 test.

Abbreviations: RVSP, right ventricular systolic pressure; LVEF, left ventricular ejection fraction; LA, left atrial; RA, right atrial.
